# Adverse pregnancy outcomes and associated risk factors among pregnant women with syphilis during 2013–2018 in Hunan, China

**DOI:** 10.3389/fmed.2023.1207248

**Published:** 2023-07-13

**Authors:** Jie Gao, Xia Chen, Min Yang, Yinglan Wu, Ting Liang, Huixia Li, Wanqin Xie

**Affiliations:** Department of Child Health Care, Hunan Provincial Maternal and Child Health Care Hospital, Changsha, Hunan Province, China

**Keywords:** pregnancy outcome, risk factor, syphilis, early detection, standard treatment

## Abstract

**Objective:**

To investigate the adverse pregnancy outcomes and associated risk factors among pregnant women with syphilis.

**Design:**

Pregnant women with syphilis in the registry for the prevention of mother-to-child transmission of AIDS, syphilis and hepatitis B in Hunan Province, China, from January 1, 2013 to December 31, 2018 were included in the study.

**Results:**

Among the 14,219 pregnant women with syphilis, 11,346 had definite pregnancy outcomes and were in singleton pregnancy. The risk factors related to adverse pregnancy outcomes include the age of pregnant women with syphilis <20 years old (aOR = 1.274, 95% CI: 1.088–1.493) or ≥ 35 years old (aOR = 1.402, 95% CI: 1.167–1.686), not married (aOR = 1.855, 95% CI: 1.453–2.367), initial syphilis detection in the late pregnancy (aOR = 1.266, 95% CI: 1.032–1.555), diagnosis of syphilis in the late pregnancy (aOR = 5.806, 95% CI: 1.796–18.770), diagnosis of syphilis during labor (aOR = 4.102, 95% CI: 1.263–13.330), husband/sexual partner infected with syphilis (aOR = 1.222, 95% CI: 1.068–1.398), untreated (aOR = 6.756, 95% CI: 5.586–8.197), and nonstandard medication (aOR = 3.300, 95% CI: 2.841–3.846).

**Conclusion:**

The prevalence of adverse pregnancy outcomes among pregnant women with syphilis in Hunan Province, China from 2013 to 2018 was relatively high. The adverse pregnancy outcomes associated with syphilis could be reduced by early detection and standard treatment of syphilis for pregnant women and their husbands/sexual partners.

## 1. Introduction

Syphilis, caused by the bacterium *Treponema pallidum* (TP), is a significant global public health problem (1, 2). The World Health Organization (WHO) estimates that there are nearly 60,000 new cases of infection worldwide annually, with the highest prevalence rate among individuals aged 15–49 years old (3). Syphilis mainly spreads through unprotected sexual contact. Additionally, it can be transmitted from untreated or inadequately treated syphilis-infected pregnant women to their babies through the placenta (in around 80% of cases), and may occur at any stage of pregnancy or disease, or occasionally through direct contact with syphilitic sores as the baby passes through the birth canal, resulting in congenital transmission. Globally, syphilis during pregnancy is the second leading cause of stillbirths and can also result in preterm birth, low birth weight, and neonatal death (4–6).

In 2007, the World Health Organization launched a global health initiative to eliminate mother-to-child transmission of syphilis (7). Due to an increase in coverage of prenatal care, screening for syphilis during pregnancy, and treatment for infected mothers, the global prevalence of syphilis among pregnant women has remained stable in recent years, and there has been a slight decrease (although still high) in the prevalence of congenital syphilis, although regional variations still exist (8, 9). According to WHO estimates, economically-disadvantaged Africa accounted for 62% of the global burden of congenital syphilis; in 2016, the rate of syphilis screening during pregnancy in that region was only 47% (10). The number of cases of congenital syphilis in Brazil increased significantly from 6,949 cases in the period from 2010 to 2015 to 19,647 cases (an increase from 240 cases to 650 cases per 100,000 live births). In 2016, the Brazilian government launched the “Syphilis No!” project. During the intervention period from 2016 to 2019, there was a monthly decrease of 21 cases per 100,000 live births in priority cities and 10 cases per 100,000 live births in non-priority cities (11). In 2020, the United States reported 2,148 cases of congenital syphilis (a rate of 57.3 cases per 100,000 live births). The ratio has been increasing every year since 2013, and there are significant racial disparities (4). Europe accounts for only 0.3% of the global burden of congenital syphilis, and from 2012 to 2016, the number of cases there decreased from an estimated 3,400 to 2,200 cases (a decrease from 30 to 19 cases per 100,000 live births) (10). Additionally, the impact of the COVID-19 pandemic in 2019 on global syphilis is still uncertain, but reduced funding, changes in outpatient environments, travel restrictions, and decreased capacity for sexual health projects may lead to a sharp increase in syphilis cases in the coming years (12, 13).

In 2014, the World Health Organization developed global guidelines that included a comprehensive process and standard for verifying mother-to-child transmission of syphilis, further standardizing the diagnosis and treatment of syphilis during pregnancy and congenital syphilis (14). Syphilis diagnosis is based on clinical appearance, direct pathogen detection (dark-field microscopy, PCR, and histology), and serological tests. Penicillin G Benzathine (BPG) is the preferred treatment for all stages of syphilis (15). However, socioeconomic factors may lead to significant differences in syphilis prevalence rates across different countries, such as Brazil, where the prevalence rate of syphilis is significantly higher due to economic, cultural, social, and territorial differences, despite having similar disease control strategies and efforts as Portugal (16). Furthermore, in the United States and many high-income countries, despite high proportions of syphilis screening and effective treatment, the prevalence of syphilis is still on the rise due to the challenges in diagnosis and testing. Symptoms of syphilis differ at different stages, and different screening algorithms and interpretations of test results may cause confusion in diagnosis. Patients may also feel shame or discomfort in seeking medical attention for sexually transmitted diseases, further adding to diagnostic difficulty (17).

In 2011, China developed a national plan to prevent mother-to-child transmission of syphilis, which was promoted nationwide in 2015. According to the Chinese Center for Disease Control and Prevention, the number of cases of congenital syphilis per 100,000 live births in China decreased from 91.6 cases to 18.4 cases between 2011 and 2018 (18). However, China’s vast territory, diverse ethnic groups, and economic and cultural differences between different regions have led to different disease distributions across the country. Hunan Province is located in central-southern China, with a population of 66 million. Since 2011, a comprehensive syphilis screening and treatment program for pregnant women has been implemented in Hunan Province. However, epidemiological characteristics of syphilis in pregnant women have not been studied by region. This study analyzes the monitoring data of the Integrated Prevention of Mother-to-Child Transmission (IPMTCT) project in Hunan Province from 2013 to 2018. The study results provide a deeper understanding of the regional prevalence of the disease and the distribution of high-risk populations, and can help develop more accurate and effective prevention strategies for the region, thereby promoting China’s overall public health level.

## 2. Materials and methods

### 2.1. Ethics statement

The study was conducted in compliance with China government’s guidelines and approved by the Medical Ethics Committee of Hunan Provincial Maternal and Child Health Care Hospital.

### 2.2. Subjects

This regional study used the registry of pregnant women infected with syphilis from the Information System of Prevention of Mother-to-Child Transmission of AIDS, Syphilis, and Hepatitis B (IPMTCT) in China, and pregnant women with syphilis in Hunan Province between January 2013 and December 2018 were included in this study. We collected information on social-demographic characteristics, syphilis screening and treatment, pregnancy outcomes, and newborn information of pregnant women through a web-based information system.

### 2.3. Screening and diagnostic criteria for syphilis in pregnant women

Under the frame of IPMTCT in China, all pregnant women received free syphilis screening by means of National Free Pre-Pregnancy Health Exams at their first visit to prenatal care. The test was either rapid plasma reagin ring card test (RPR) or toluidine red unheated serum test (TRUST). The patients with positive results in RPR or TRUST were confirmed in the TPAS test by using *T. pallidum* particle assay (TPPA), *T. pallidum* haemagglutina-tion assay (TPHA), or enzyme-linked immunosorbent assay (ELISA). Syphilis infection was diagnosed as both NTPAS test (RPR or TRUST) and TPAS test (TPPA or TPHA or ELISA) being positive. According to Gestational Syphilis and Congenital Syphilis Prevention and Treatment Guideline (19, 20), syphilis infection was classified into the latent, primary, secondary, and tertiary stages.

### 2.4. Treatment for syphilis in pregnant women

Benzathine penicillin G (2.4 million units) is given as a once-weekly injection for 3 weeks, or procaine penicillin G (800,000 units) is given as a daily injection for 15 days as the first choice for treating syphilis in pregnant women. For those allergic to penicillin, ceftriaxone (1 g daily) for 10 days or erythromycin (500 mg four times daily) for 15 days are recommended. Treatment status during pregnancy is defined as adequate treatment or inadequate or no treatment. Adequate treatment is defined as completing at least one course of treatment for at least 30 days before delivery. Women who complete less than one course of treatment are defined as untreated or inadequately treated. Women with a relapse or reinfection should be retreated (21).

### 2.5. Congenital syphilis

Any of the following conditions in a newborn are defined as congenital syphilis: ① *Treponema pallidum* is detected by dark field microscopy (or silver staining) in skin mucosal lesions or tissue specimens of children. ② The test of *Treponema pallidum* IgM antibody is positive. ③ The serologic test of non-*Treponema pallidum* antigen at birth is positive, with a titer being or more than four times as many as that of the mother before delivery, and the serologic test of *Treponema pallidum* antigen is positive. ④ The newborn baby has more than two clinical features and manifestations as follows: acral palmetto and toe desquamation, macula, mucous membrane damage, hepatosplenomegaly, pathological jaundice, low weight, dyspnea, peritoneal effusion, edema, syphilis pseudopalsy, anemia, and thrombocytopenia (22).

### 2.6. Adverse pregnancy outcomes

Any of the following conditions in a newborn are defined as APO: ① The pregnancy is terminated at less than 28 weeks and the fetus weighs less than 1,000 g. ② Intrauterine death of an embryo happens at ≤28 gestational weeks. ③ A dead fetus is delivered after 28 weeks of gestation. ④ Delivery happens between 28 and 37 weeks of gestation. The newborn baby at this time is called premature infant. ⑤ The newborn baby dies within 1 month of birth. ⑥ Birth weight is less than 2.5 kg. In a word, as a composite outcome, adverse pregnancy outcome is defined as any of the occurrence of miscarriage, preterm birth, low birth weight, dead fetus, stillbirth, neonatal death, or neonatal congenital syphilis.

### 2.7. Statistical analysis

Data were entered and cleaned on EpiData 3.1 (JensM.Lauritsen, Michael Bruus, and Mark Myatt, Odense, Denmark) and analyzed on SPSS 24.0 (IBM, Chicago, IL, United States). Categorical variables were compared using the Chi-square test. Multivariate Logistic regression analysis was performed with adverse pregnancy outcomes as the dependent variable and general epidemiological characteristics and relevant clinical characteristics of syphilis-infected pregnant women as independent variables, and odds ratios (A OR) and 95% confidence intervals (CI) were calculated and adjusted. The significant level was *p* < 0.05.

## 3. Results

### 3.1. Positive syphilis rates among pregnant women are on the rise from 2013 to 2018

**Figure 1:**
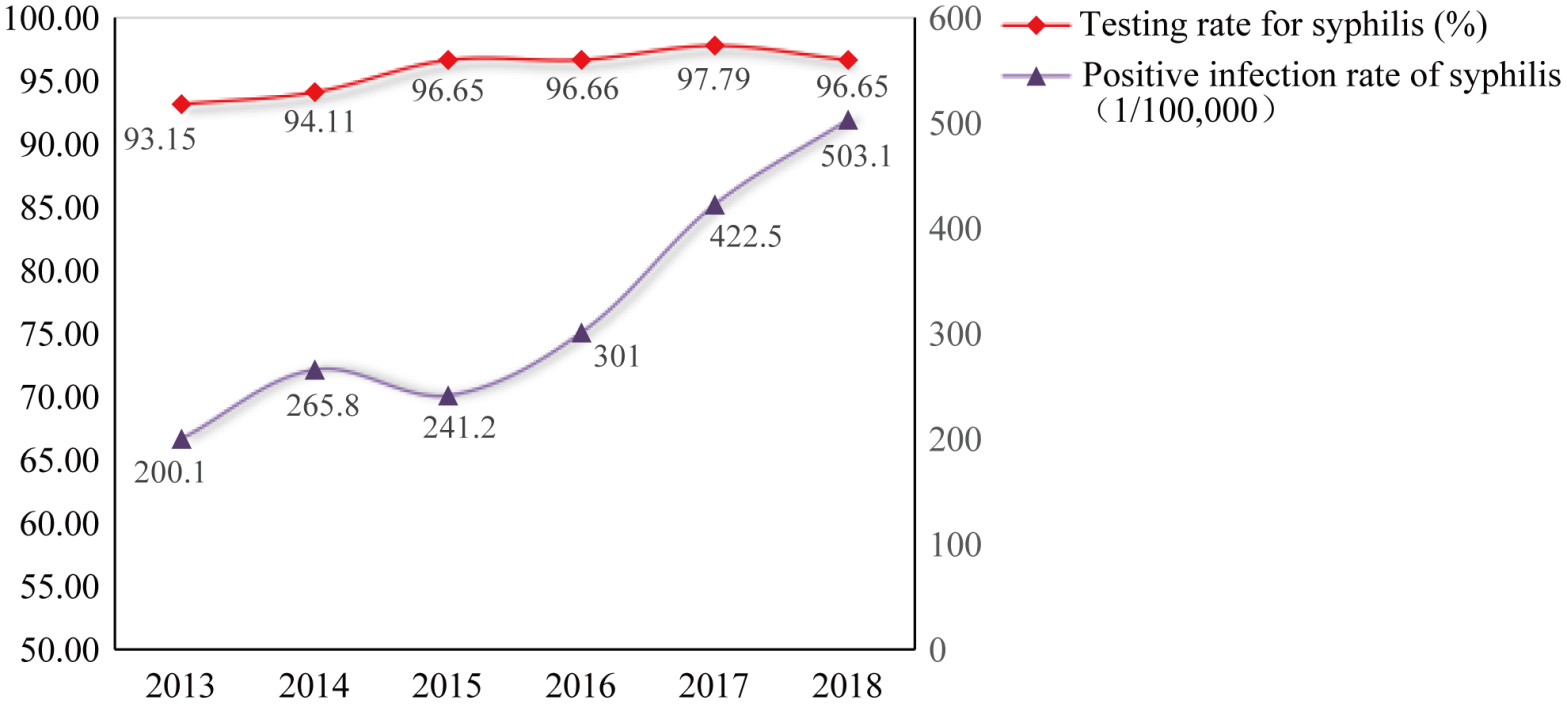
Syphilis testing rate and infection rate among pregnant women in Hunan Province from 2013 to 2018.

A total of 4,458,696 pregnant women in Hunan Province, China from 2013 to 2018 received syphilis screening, with an average annual coverage rate of 95.78%. A total of 14,219 cases of pregnant women with syphilis were reported, and the average annual positive rate of syphilis among pregnant women was 318.9/100,000. It is notable that there was an upward trend for syphilis positive rate among pregnant women during the period, rising from 200.1/100,000 in 2013 to 503.1/100,000 in 2018 (Figure 1).

### 3.2. Demographic and diagnostic characteristics of pregnant women with syphilis

The average age of pregnant women with syphilis in Hunan Province from 2013 to 2018 is 29.51 ± 0.05 years (range: 14–53 years). In the cohort, housewives and unemployed women account for 50.1% (7,124 cases), whereas farmers account for 32.8% (4,664 cases). 96.0% (13,647 cases) of the women are married (including first marriage, in-marriage, and cohabitation). Women with senior middle school, junior middle school or below, and primary school education account for 88.1% (12,526 cases). The prevalence of recessive syphilis is 69.8% (9,925 cases), primary syphilis is 4.1% (583 cases), secondary syphilis is 0.8% (114 cases), tertiary syphilis is 0.4% (57 cases), and unknown syphilis is 24.9% (3,540 cases). 81.4% (11,574 cases) of syphilis are diagnosed during pregnancy, 18.1% (2,574 cases) are diagnosed during or after delivery, and 0.5% (71 cases) belong to previous diagnosis of syphilis.

### 3.3. Pregnancy outcomes and influencing factors of pregnant women with syphilis

Among 14,219 cases of pregnant women with syphilis, there are 1,027 cases of artificial termination of pregnancy, 202 cases of multiple pregnancies, 1,644 cases of unknown pregnancy outcomes or lost follow-up, and 11,346 cases of singleton pregnancy with definite pregnancy outcomes, including spontaneous abortion, dead fetus or stillbirth, premature birth or low birth weight, neonatal asphyxia, neonatal pneumonia, birth defects, and congenital syphilis. Of the 11,346 cases, the total prevalence of adverse pregnancy outcomes is 19.1% (2,166 cases), and the details are shown in Table 1.

**Table 1:** The detailed adverse outcomes of singleton pregnancies among 11,346 women with syphilis.

Adverse pregnancy outcomes	Number	%
Spontaneous abortion	222	2
Dead fetus or stillbirth	133	1.2
The newborn who died within 7 days of birth	75	0.7
Premature birth or low birth weight	1,221	10.8
Neonatal asphyxia	121	1.1
Neonatal pneumonia	87	0.9
Birth defects	161	1.4
Congenital syphilis	146	1.3
Total	2,166	19.1

The same newborn may have multiple adverse pregnancy outcomes.

The syphilis infected pregnant women are divided into the normal pregnancy group (9,943 cases) and the adverse pregnancy group (1,403 cases) based on no adverse pregnancy outcome or any adverse pregnancy outcome. Chi-square tests show that there are differences in age, education level, marital status, gestational week of initial test, gestational week at diagnosis of syphilis infection, previous diagnosis of syphilis, current syphilis status of husband/sexual partners, and standardized treatment between the normal pregnancy group and the adverse outcome group in pregnant woman with syphilis (values of *p* < 0.05, as shown in Table 2). There are no statistically significant differences in ethnicity, occupation, gravidity, parity, adverse pregnancy history, syphilis-infection stage, and the route of syphilis transmission between the normal pregnancy group and the adverse outcome group in pregnant woman with syphilis (All values of *p* ≥ 0.05, Table 2).

**Table 2:** Comparison of demographic characteristics, maternal history, and syphilitic related characteristics of pregnant women with syphilis.

Characteristics	Normal group (*n* = 9,943)	Adverse pregnancy outcome group (*n* = 1,403)	*X^2^*	*p*
*Maternal age, n (%)*			21.762	<0.001
<20 years	268(2.7)	52(3.7)		
20–34 years	7,702(77.5)	1,009(71.9)		
≥35 years	1973(19.8)	342(24.4)		
*Ethnicity, n (%)*			3.818	0.051
Han	8,683(87.3)	1,199(85.5)		
Minorities	1,260(12.7)	204(14.5)		
*Education level, n (%)*			9.218	0.027
Primary school or below	741(7.5)	125(9)		
Middle-school (junior middle-school, senior middle school)	8,102(81.4)	1,109(79)		
Technical secondary school, college, university, or above	664(6.7)	89(6.3)		
Unknown	436(4.4)	80(5.7)		
*Occupation, n (%)*			4.847	0.089
Farmer	3,529(35.5)	456(32.5)		
Housewife or unemployed	4,883(49.1)	723(51.5)		
Others	1,531(15.4)	224(16)		
*Marriage, n (%)*			35.644	<0.001
Married	9,572(96.3)	1,303(92.8)		
Unmarried	371(3.7)	100(7.2)		
*Gravidity, n (%)*			3.037	0.219
1 previous pregnancy	1919(19.3)	254(18.1)		
2 previous pregnancies	3,123(31.4)	423(30.1)		
≥3 previous pregnancies	4,901(49.3)	726(51.8)		
*Parity, n (%)*			0.13	0.719
Primipara	3,184(32.0)	456(32.5)		
Pluripara	6,759(68.0)	947(67.5)		
*Adverse pregnancy and birth history, n (%)*			2.126	0.145
No	7,610(76.5)	1,049(74.8)		
Yes	2,333(23.5)	354(25.2)		
*Gestational week of initial test, n (%)*			7.591	0.022
Early stage	6,317(63.5)	923(65.8)		
Middle stage	2,634(26.5)	325(23.2)		
Late stage	992(10.0)	155(11)		
*Syphilis previously diagnosed, n (%)*			5.957	0.015
No	6,627(66.6)	981(69.9)		
Yes	3,316(33.4)	422(30.1)		
*Gestational week of syphilis confirmation this time, n (%)*			20.962	<0.001
Early or middle stage	5,965(60.0)	818(58.3)		
Late stage	1744(17.5)	225(16)		
At delivery	1736(17.5)	262(18.7)		
After delivery	412(4.1)	86(6.1)		
Others	86(0.9)	12(0.9)		
*The diagnosis of syphilis stage, n (%)*			0.468	0.791
Recessive syphilis	6,854(68.9)	977(69.6)		
Stages I–III	518(5.2)	75(5.3)		
Unspecified	2,571(25.9)	351(25)		
*Route of infection, n (%)*			9.295	0.054
Sex transmission	3,040(30.6)	381(27.2)		
Blood transmission	81(0.8)	17(1.2)		
Mother-to-fetus transmission	28(0.3)	5(0.4)		
Others transmission (e.g., skin-to-skin and indirect)	39(0.4)	4(0.3)		
Unknown	6,755(67.9)	996(71)		
*Current syphilis status of husbands or sexual partners, n (%)*			22.915	<0.001
Positive	616(6.2)	80(5.7)		
Negative	3,222(32.4)	370(26.4)		
Unknown or untested	6,105(61.4)	953(67.9)		
*Treatment, n (%)*			438.73	<0.001
Standard	3,617(36.4)	270(19.2)		
Non-standard	5,232(52.6)	712(50.7)		
Untreated	1,094(11.0)	421(30)		

### 3.4. The gestational week of initial test and non-standardized treatment are the most relevant factors for adverse pregnancy outcomes

Multifactor logistic regression analysis show that the pregnant women with syphilis, who are<20 years old (aOR = 1.274, 95% CI: 1.088–1.493) or ≥ 35 years old (aOR = 1.402, 95% CI: 1.167–1.686), not married (aOR = 1.855, 95% CI: 1.453–2.367), initially tested in the third trimester (aOR = 1.266, 95% CI: 1.032–1.555), confirmed to be infected by syphilis in the third trimester/at delivery (aOR = 5.806, 95% CI: 1.796–18.770), with husband/sexual partner infected by syphilis (aOR = 1.222, 95% CI: 1.068–1.398), and (or) not treated (aOR = 6.756, 95% CI:5.586–8.197)/in standard treatment (aOR = 3.300, 95% CI:2.841–3.846), are more likely to have adverse pregnancy outcomes as shown in Table 3.

**Table 3:** Multi-factor Logistic regression analysis of adverse pregnancy outcomes of pregnant women with syphilis.

Influencing factors	B	*p*	aOR	aOR (95%CI)
Age/year				
<20	0.242	0.003	1.274	1.088–1.493
20–34	-	0.000	1.000	-
≥35	0.338	0.000	1.402	1.167–1.686
Marriage				
Married	-	0.000	1.000	-
Unmarried	0.618	0.000	1.855	1.453–2.367
Gestational week of initial test				
Early stage	-	0.023	1.000	-
Middle stage	0.067	0.472	0.935	0.890–1.284
Late stage	0.236	0.024	1.266	1.032–1.555
Gestational week of syphilis confirmation this time				
Early or middle stage	-	0.000	1.000	-
Late stage	1.759	0.003	5.806	1.796–18.770
At delivery	1.412	0.019	4.102	1.263–13.330
After delivery	1.148	0.056	3.152	0.971–10.234
Others	1.311	0.032	3.709	1.123–12.251
Current syphilis status of husbands or sexual partners				
Positive	-	0.013	1.000	-
Negative	0.203	0.003	1.222	1.068–1.398
Unknown or untested	0.064	0.131	1.066	0.825–1.377
Treatment				
Standard	-	0.000	1.000	-
Non-standard	1.196	0.000	3.301	2.841–3.846
Untreated	1.912	0.000	6.756	5.586–8.197

## 4. Discussion

Vertical transmission of syphilis results in adverse birth outcomes, including fetal death (stillbirth), neonatal death, preterm birth, a low birth weight, and congenital infection (23). Findings from the present study show that while the coverage rate of syphilis detection among pregnant women in Hunan Province, China was high and continuously increased (from 93.15 to 96.65%) during the period of 2013–2018, the positive rate of syphilis in pregnant women had more than doubled (from 214.9/100,000 to 475.2/100,000). This could be due to the more timely surveillance by the IPMTCT program, and more probably, the increased awareness of syphilis detection in pregnant women who had high-risk behaviors.

The rate of adverse pregnancy outcomes (APOs) among pregnant women with syphilis is one of the effective indicators for evaluation of mother-to-child blocking of syphilis. The present study shows that the total prevalence of APOs among pregnant women with syphilis in Hunan Province, China from 2013 to 2018 was lower than the prevalence of APOs in untreated syphilis-infected pregnant women in the meta-analysis by Qin et al. (24) (19.1 vs. 76.8%). However, compared with developed region such as Zhejiang Province, China, which reported a rate of 13.02% (25) of APOs among pregnant women with syphilis between 2013 and 2014, the quality of intervention for syphilis in pregnant women in Hunan Province needs to be strengthened.

Syphilis detection in early pregnancy enables the standardized treatment of syphilis for pregnant women as early as possible, which is one of the important means to prevent congenital syphilis (26). Our study shows that the later a pregnant woman tested for syphilis, the higher the risk of adverse pregnancy outcomes. Women diagnosed in the third trimester and at delivery had a higher risk of APOs than women diagnosed in the first and second trimesters. However, due to factors such as the testing capacity of some health institutions, the limited knowledge and skills of medical staff, and inadequate health education for pregnant women, the proportion of pregnant women who are tested for syphilis in early pregnancy is still low, which affects subsequent treatment.

Penicillin is the first choice of treatment for pregnant women with syphilis infection. Early detection, early treatment, and whole course standard treatment are the key to prevent congenital syphilis (27). Many studies have shown that regular penicillin treatment for syphilis-infected pregnant women is effective in reducing APOs and the prevalence of congenital syphilis (28, 29). The present study shows that compared to the standardized treatment, non-standard treatment, or no treatment for pregnant women with syphilis were more likely to have APOs. There are possible two reasons for the non-standard use of drugs: on the one hand, a part of the healthcare practioners are not proficient in the standard treatment of pregnant women with syphilis, on the other hand, although antibiotic treatment for syphilis during pregnancy is a low-cost and safe effective method, some pregnant women in China instinctively believe that antibiotics have adverse effects on the fetus. This situation may involve multiple reasons, including concerns about the drug’s adverse effects on the fetus among some pregnant women. In addition, some pregnant women may not fully understand the necessity and importance of antibiotic treatment and may believe that taking antibiotics will have adverse effects on maternal and infant health. Furthermore, although antibiotic prescriptions in the Chinese medical system require the approval and review of doctors, some pregnant women may purchase antibiotics without consulting or being guided by doctors, leading to irrational use and abuse of antibiotics. To improve the knowledge and understanding of Chinese pregnant women regarding the use of antibiotics and their resistance, it is necessary to strengthen medical and health education, promote related knowledge, establish scientific and reasonable medication guidance and regulatory systems, and also enhance the knowledge training of medical staff regarding the diagnosis, treatment, and prevention of syphilis during pregnancy.

According to the estimated time of syphilis infection and the type of symptoms exhibited by the patient, syphilis can be divided into four stages. The transmission of *Treponema pallidum* is highest in early syphilis, with approximately 30% of people becoming infected with syphilis after having sexual contact with early syphilis patients (30, 31). This study suggests that the infection status of husband/sexual partners is one of the factors affecting the APOs of pregnant women with syphilis. Sexual transmission is the main approach of syphilis transmission. Even after standardized treatment, there is a risk of re-infection by the husband/partners for pregnant women with syphilis (32, 33). Maternal fear of domestic violence, poor communication between partners, and stigma related to syphilis are the main obstacles leading to non-detection of sexual partners (34). A study conducted in Shenzhen, China, indicated that partner infection had a large effect on pregnancy outcomes, with an estimated OR of 2.02 (24). Lack of standardized treatment for the sexual partners may expose the pregnant women to recurrent or persistent infection. This is why the detection and treatment of husband/sexual partners are also a priority in the prevention of APOs associated with syphilis in pregnancy. It should be noted that primary syphilis in males typically presents as a single, painless, clean-based ulcer at the site of inoculation of *Treponema Pallidum*, accompanied by regional lymphadenopathy, and most commonly occurs in the genital and anal regions (17). Primary syphilis may be misdiagnosed as other ulcerative lesions. If the symptoms are painless and the infection occurs on hard-to-observe surfaces such as the anal region or testicles, the syphilis diagnosis may be delayed or missed. Patients carrying syphilis, although the course of the disease can progress to other stages, chancres generally only last for a few weeks and can heal spontaneously even without treatment (35, 36). Serological tests, especially lipid tests, in this stage may be non-reactive and yield false-negative results; therefore, a high clinical suspicion is crucial for initiating empirical treatment and reducing the risk of transmission and progression to later stages.

Findings from the present study show that pregnant women with syphilis younger than 20 years or older than 35 years are more likely to have adverse pregnancy outcomes. Bai et al. (37) observed that there is a statistical difference in age distribution between normal pregnancy outcomes and adverse pregnancy outcomes of gestational syphilis. The study of Dou et al. (38) also showed that syphilis infected pregnant women younger than 20 years are more likely to have adverse pregnancy outcomes compared with those between 20 and 34 years old. However, a previous study has shown that there is no statistical difference of adverse pregnancy outcomes such as spontaneous abortion and dead fetus between the age groups of pregnant women with syphilis (39). Therefore, further investigations to address the inconsistent findings of these studies are anticipated.

There are some limitations in the study. The use of data solely from the national health information registration system in syphilis epidemiological research may be limited by the following factors: ① The data source is limited to the country’s health information system, which may result in unrecorded cases and transmission data. ② Syphilis patients may choose not to report to the health information system or fail to be diagnosed for various reasons. This may lead to underestimation of disease prevalence and transmission. ③ Different regions, age and gender groups, and transmission routes may have different syphilis infection risks, which may not be fully recorded and reflected in the health information system. Therefore, there may be certain biases in using a single national health information system to study syphilis epidemiology. There is a lack of specific collection and analysis for risk factors associated with certain adverse pregnancy outcomes. Moreover, the research results are susceptible to selection bias, information bias, and confounding bias. Thirdly, the data collection for congenital syphilis babies only includes the cases with clear diagnosis, which may underestimate the regional prevalence of congenital syphilis.

## 5. Conclusion

Despite a commitment to the national strategy for syphilis screening and comprehensive intervention in pregnant women in China since 2011, many pregnant women with syphilis have not been timely diagnosed and treated. To eliminate congenital syphilis, early detection and standard treatment of syphilis for pregnant women and their husbands/sexual partners must be improved.

## Data availability statement

The data analyzed in this study are subject to the following licenses/restrictions: datasets generated and/or analyzed in the present study are available from the corresponding author upon reasonable request. Requests to access these datasets should be directed to YW, 275513435@qq.com.

## Ethics statement

The study was conducted in compliance with China government’s guidelines and approved by the Medical Ethics Committee of Hunan Provincial Maternal and Child Health Care Hospital.

## Author contributions

JG conceptualized and conducted the study, analyzed the data, and drafted successive versions of the manuscript. YW provided inputs to the planning of the study and guided all steps of the study process. XC managed and oversaw the administrative and field data collection processes. MY managed data entry and cleaning. TL, HL, and WX provided inputs for the planning of the study and revised successive drafts of the manuscript. All authors contributed to the article and approved the submitted version.

## Funding

This work was supported by Health Commission of Hunan Province (D202312037668).

## Conflict of interest

The authors declare that the research was conducted in the absence of any commercial or financial relationships that could be construed as a potential conflict of interest.

## Publisher’s note

All claims expressed in this article are solely those of the authors and do not necessarily represent those of their affiliated organizations, or those of the publisher, the editors and the reviewers. Any product that may be evaluated in this article, or claim that may be made by its manufacturer, is not guaranteed or endorsed by the publisher.
